# VERMONT non-optimised: Feasibility of angiography-derived vFFR using baseline diagnostic catheter images

**DOI:** 10.1016/j.ijcha.2026.101905

**Published:** 2026-03-19

**Authors:** Daniel Akrawi, Krishna Kadappu, James Xu, Tamer Yousef Naguib Badie, Oliver Gibbs, Hashim Kachwalla, Phong TD Nguyen, Rahul Kurup, Upul Premawardhana, Sidney Lo, Justyn Huang, Hao Tran, Kavie Soosapilla, Aiden O’loughlin, Annemarie Hennessy, Giuseppe Femia

**Affiliations:** a Department of Cardiology, Campbelltown Hospital, Sydney, NSW, Australia; b Department of Cardiology, Liverpool Hospital, Sydney, NSW, Australia; c School of Medicine, Western Sydney University, Campbelltown, NSW, Australia; d Faculty of Medicine, University of New South Wales, Liverpool, NSW, Australia; e Faculty of Medicine and Health, University of Sydney, Westmead, NSW, Australia; f Faculty of Medicine and Health, University of Sydney, Camperdown, NSW, Australia

**Keywords:** Fractional flow reserve, Vessel fractional flow reserve, Angiogram based fractional flow reserve, FFR, vFFR, Baseline diagnostic images

## Abstract

**Background:**

Vessel-fractional-flow-reserve (vFFR) estimates coronary physiology from the three-dimensional reconstruction of two angiographic projections using computational fluid dynamics. Although its diagnostic accuracy using optimised angiographic acquisitions is well established, evidence supporting its use with baseline diagnostic catheter images remains limited.

**Aims:**

To evaluate the diagnostic performance of real-time vFFR derived from baseline diagnostic catheter images against wire-based FFR, and to compare its performance with vFFR computed from optimised angiographic projections.

**Methods:**

VERMONT Non-Optimised was a prospective, single-centre, blinded study in which real-time vFFR derived from both baseline diagnostic and optimised images were measured and compared with simultaneous wire-based FFR. A wire-based FFR of ≤ 0.80 defined a physiologically significant lesion.

**Results:**

In 195 patients with 205 intermediate lesions, 56 (27.3%) lesions were excluded from vFFR analysis. vFFR derived from baseline diagnostic images demonstrated an AUC of 0.91 (95% CI,0.87–0.96) for detecting lesions with FFR ≤ 0.80, achieving 94% sensitivity, 75% specificity, a negative predictive value of 96%, and a positive predictive value of 67%. Baseline diagnostic and optimised vFFR were strongly correlated (R = 0.87,p < 0.001), with a mean bias of −0.0075 ± 0.0490 and an intraclass correlation coefficient of 0.93 (95% CI,0.90–0.95), indicating excellent agreement.

**Conclusion:**

Real-time vFFR derived from judiciously selected baseline diagnostic catheter images demonstrated strong overall accuracy and high sensitivity for detecting physiologically significant lesions, with similar diagnostic performance to vFFR derived from optimised images. These findings support the use of vFFR as a reliable screening tool for intermediate lesions in both prospective and retrospective settings.

## 1 Introduction

Angiography-based fractional flow reserve (FFR) techniques have emerged as a promising alternative to wire-based FFR, enabling functional lesion assessment without pressure wires or pharmacological hyperaemia [1], [2], [3]. Vessel fractional flow reserve (vFFR) is an angiography-derived physiology platform that reconstructs the coronary artery in three dimensions from two cineangiographic projections and applies computational fluid dynamics to estimate the trans-stenosis pressure gradient [4], [5], [6]. A fundamental limitation of angiogram-derived physiology is its dependence on image quality, as accurate vessel reconstruction and haemodynamic modelling require precise delineation of coronary geometry [4]. Accordingly, current recommendations advocate the acquisition of additional optimised angiographic views, typically ≥15 frames per second, with minimal vessel overlap or foreshortening, no table movement, and adequate contrast opacification, often obtained after routine diagnostic angiography [7], [8]. Whether baseline diagnostic catheter images alone are sufficient for reliable vFFR computation remains uncertain.

We recently published the VERMONT study, a single-centre, blinded, prospective observational investigation evaluating real-time vFFR against wire-based FFR ≤ 0.80 using additional optimised angiographic projections acquired specifically for vFFR analysis [7]. Real-time vFFR demonstrated excellent diagnostic accuracy (AUC 0.92), with high sensitivity (90%) and negative predictive value (93%), supporting its role as a reliable physiological screening tool for intermediate coronary stenosis [7].

A key unresolved question is whether vFFR can be reliably computed from baseline diagnostic catheter images alone, defined as the routinely acquired, non-optimised cineangiographic projections obtained during initial diagnostic coronary angiography, prior to any protocol-mandated optimisation. If feasible, this approach could eliminate the need for additional image acquisition, reduce contrast and radiation exposure, shorten procedural time, and enable retrospective vFFR assessment from existing angiographic datasets.

## 2 Methods

### 2.1 Study design and population

The VERMONT (VEssel ffR assessMent Of steNosis severiTy) Non-Optimised study was a pre-specified subgroup analysis derived from the same prospectively enrolled cohort as the original VERMONT study. It was an investigator-initiated, single-centre, blinded, prospective observational study designed to evaluate the diagnostic performance of real-time vessel fractional flow reserve (vFFR) derived exclusively from baseline diagnostic catheter images, using wire-based FFR ≤ 0.80 as the reference standard for physiological significance. It also aimed to directly compare these results with vFFR computed from optimised angiographic projections acquired according to the original VERMONT protocol [7]. The study was approved by the South-West-Sydney Local Health District human research ethics committee (study number: 2021/ETH12209) and was conducted in accordance with the National Statement on Ethical Conduct in Human Research and the Ethical Guidelines of the 1975 Declaration of Helsinki. A full waiver of consent was approved for the study.

All patients with angiographically intermediate coronary artery disease (50–70% stenosis) who underwent wire-based FFR at Campbelltown Hospital (Sydney, Australia) between February 2022 and August 2023 were eligible for inclusion, provided that adequate optimised cineangiographic images were available for vFFR computation. Wire-based FFR was performed either during diagnostic angiography or prior to planned percutaneous coronary intervention (PCI) to assess the functional significance of the target lesions.

Lesions were excluded from analysis if baseline diagnostic catheter images displayed extensive vessel overlap, foreshortening, poor contrast opacification, excessive panning or inadequate angle projections [See Appendix A for definitions]. Exclusion decisions were made by consensus between two investigators (DA and the interventionalist) prior to vFFR analysis. All other lesions were included in the analysis.

### 2.2 Study procedures

All patients initially had a baseline coronary angiogram with diagnostic catheters, with image acquisition parameters determined by the treating interventional cardiologist according to routine clinical practice. Patients with intermediate coronary artery lesions selected for physiological assessment by the interventional cardiologist underwent both wire-based FFR and vFFR during the same procedure as previously described [7]. After recording the aortic root pressure, the coronary artery was engaged with a guide catheter, and intracoronary glyceryl trinitrate (200 mcg) was administered. At least two optimised angiographic projections were then obtained ≥30 degrees apart, at 15 frames per second and at a magnification of 25 cm, with no table movement. In cases of significant vessel overlap, foreshortening, or suboptimal contrast opacification, further acquisitions were performed as required to secure two optimal frames for vFFR analysis. A pressure wire (PressureWire X; Abbott Laboratories, Abbott Park, IL, USA) was advanced across the lesion, and FFR was measured according to guidelines, with maximal blood flow induced by intravenous adenosine infusion (140 mcg/kg/min).

Wire-based FFR and vFFR were performed independently and simultaneously, with both the interventionalist and the vFFR operators blinded to each other’s results. vFFR analysis was performed using CAAS Workstation 8.2 (Pie Medical Imaging). vFFR derived from optimised angiographic images was first computed online during the procedure by the primary investigator (DA). Immediately thereafter, during the same procedure, a second blinded investigator calculated vFFR using the baseline diagnostic catheter images. All images were analysed at their native frame rate without the use of temporal interpolation algorithms. Decisions regarding case exclusion were made by consensus between two investigators (DA and the interventionalist) prior to initiating vFFR analysis. A threshold of FFR ≤ 0.80 was applied to define a physiologically significant lesion for all analyses. Clinical decision making was based on wire-based FFR and not vFFR.

Lesions were categorised as focal (<20 mm), diffuse (≥20 mm), ostial (within 3 mm of the vessel origin), or moderately to severely calcified (visible calcium on angiography prior to contrast).

### 2.3 Study endpoints

The primary endpoint was the diagnostic performance of real-time vFFR derived from baseline diagnostic catheter images compared to wire-based FFR in the assessment of intermediate coronary lesions, and compared to vFFR derived from optimised images. Haemodynamic significance was defined as a wire-based FFR of ≤ 0.80.

### 2.4 Statistical analysis

The normality of continuous variables was assessed through visual inspection of histograms and the Shapiro-Wilk test. Variables following a normal distribution are presented as a mean ± standard deviation (SD), while those with non-normal distributions are reported as medians with interquartile ranges (25th–75th percentile). Categorical variables are summarized as frequencies and percentages. The relationship between baseline diagnostic vFFR, optimised vFFR and wire-based FFR were illustrated using scatter plots and quantified using Pearson’s correlation coefficients (r). The strength of correlation was interpreted as follows: 0.10–0.39 weak, 0.40–0.69 moderate, 0.70–0.89 strong, and 0.90–1.00 very strong [9]. The agreement between baseline diagnostic vFFR, optimised vFFR and wire-based FFR were evaluated using Bland-Altman plots with corresponding 95% limits of agreement. The intraclass correlation coefficient (ICC) was used to assess the agreement between baseline non-optimised and optimised vFFR, and was interpreted as: <0.50 poor, 0.50–0.75 moderate, 0.75–0.90 good, and >0.90 excellent reliability [10].

The diagnostic performance of baseline diagnostic and optimised vFFR in detecting a wire-based FFR ≤ 0.80 was evaluated by generating receiver operating characteristic (ROC) curves, calculating the area under the curve (AUC), and comparing AUCs with DeLong’s test. Sensitivity, specificity, positive predictive value (PPV), and negative predictive value (NPV) of vFFR ≤ 0.80 for predicting wire-based FFR ≤ 0.80 were also determined. Demographic characteristics, cardiovascular risk factors, and lesion characteristics were compared between false-positive (FP) and true-negative (TN) cases for baseline diagnostic vFFR. Continuous variables were analysed using the independent samples *t*-test for normally distributed data and the Mann–Whitney *U* test for non-parametric data. Categorical variables were compared using the chi-square test. A p value < 0.05 was considered statistically significant. All statistical analyses were conducted using SPSS Statistics, Version 30 (IBM Corp., Armonk, NY, USA). Unless stated otherwise, p-values are two-sided, with values less than 0.05 considered statistically significant.

## 3 Results

### 3.1 Enrolment

**Fig. 1:**
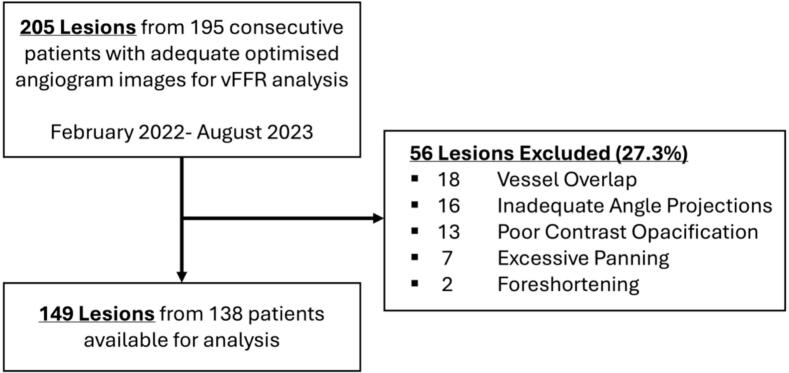
Flow chart of patient inclusion and exclusion.

A total of 195 patients with 205 lesions were enrolled, all of whom had adequate optimised angiographic images for vFFR analysis. On review of baseline diagnostic catheter images, 56 lesions (27.3%) were excluded due to vessel overlap (n = 18), inadequate angle projections (n = 16), poor contrast opacification (n = 13), excessive panning (n = 7), or foreshortening (n = 2) **[**Fig. 1**].** The final analysis therefore included 138 patients with 149 lesions.

### 3.2 Baseline characteristics and procedural data

Baseline characteristics and procedural data are listed in Table 1**.** The median age was 65 years (IQR 59–73), and 98 (71%) were male. The mean body-mass-index (BMI) was 29 ± 6 kg/m^2^ and 97 (70%) were referred for stable angina. Overall, 108 (78%) patients had hypertension, 116 (84%) had dyslipidaemia, 52 (38%) had diabetes and 71 (52%) were either current or ex-smokers. The target vessels were the left-anterior-descending/ diagonal arteries (79%), the right-coronary-artery (13%) and the left-circumflex/obtuse-marginal arteries (8%). Coronary lesions were characterised as being diffuse (62%), focal (38%), bifurcation (15%), ostial (10%) and moderate or severely calcified (23%). The mean diameter stenosis was 44 ± 11%, lesion length was 23 ± 16 mm and minimal lumen diameter was 1.6 ± 0.4 mm. Among the baseline diagnostic catheter images, 59 (40%) were acquired at 7 frames per second (fps), 9 (6%) at 10 fps, 51 (34%) at 15 fps, and 30 (20%) at 30 fps**.**

**Table 1:** Baseline Characteristics and Procedural Data.

**Demographics**	**Total N = 138 patients**
Age, years, median (IQR)	65 (59–73)
Male gender, n (%)	98 (71)
Body Mass Index, kg/m^2^, mean ± SD	29 ± 6

**Cardiovascular Risk Factors, n (%)**	
Hypertension	108 (78)
Dyslipidaemia	116 (84)
Type 2 Diabetes	52 (38)
Current Smoker	19 (14)
Ex-Smoker	52 (38)
Non-Smoker	66 (48)
Family History of Ischaemic Heart Disease	33 (24)
Peripheral Vascular Disease	2 (1)
Cerebrovascular Accident	11 (8)
Previous Myocardial Infarction	12 (9)
Previous Percutaneous Coronary Intervention	26 (19)
Previous Percutaneous Coronary Intervention to same vessel as FFR	10 (7)

**Pathology Results, mean ± SD**	
eGFR, ml/min	75 (15)
Haemoglobin, g/L	140 (16)

**Coronary Angiography Indication, n (%)**	
Stable Angina	97 (70)
Unstable Angina	18 (13)
Non-ST Elevation Myocardial Infarction	23 (17)

**Lesion Location, n (%)**	**Total N = 149 lesions**
Left Anterior Descending Artery /Diagonal Artery	117 (79)
Right Coronary Artery	20 (13)
Left Circumflex Artery/Obtuse Marginal Artery	12 (8)

**Lesion Characteristics, n (%)**	
Focal lesion (< 20 mm)	56 (38)
Diffuse lesion (**≥**20 mm)	93 (62)
Bifurcation lesion	23 (15)
Ostial lesion	15 (10)
Tortuous lesion	6 (4)
Moderate or severe calcification	34 (23)

**3D Quantitative Coronary Angiography, mean ± SD**	
Lesion Length, mm	23 ± 16
Minimal Luminal Diameter, mm	1.6 ± 0.4
Minimal Luminal Area, mm^2^	2.1 ± 1.0
Diameter stenosis %	44 ± 11
Reference vessel diameter, mm	2.9 ± 0.6

**FFR Indices**	
Wire-based FFR, mean ± SD; median (IQR)	0.82 ± 0.08; 0.82 (0.77–0.87)
Baseline Diagnostic vFFR, mean ± SD; median (IQR)	0.79 ± 0.10; 0.81 (0.74–0.86)
Optimised vFFR, mean ± SD; median (IQR)	0.80 ± 0.09; 0.81 (0.75–0.86)
Wire-based FFR ≤ 0.80, n (%)	52 (35%)
Baseline Diagnostic vFFR ≤ 0.80, n (%)	73 (49%)
Optimised vFFR ≤ 0.80, n (%)	70 (47%)

**Abbreviations:** eGFR: Estimated Glomerular Filtration Rate, FFR: Fractional Flow Reserve, IQR: Interquartile Range, SD: Standard Deviation, vFFR: Vessel Fractional Flow Reserve.

### 3.3 Correlation and diagnostic performance

#### 3.3.1 Wire-based FFR

The mean wire-based FFR was 0.82 ± 0.08 [Table 1], with values ≤ 0.80 observed in 35% of measurements **[**Table 1**].**

#### 3.3.2 Baseline diagnostic vFFR

**Fig. 2:**
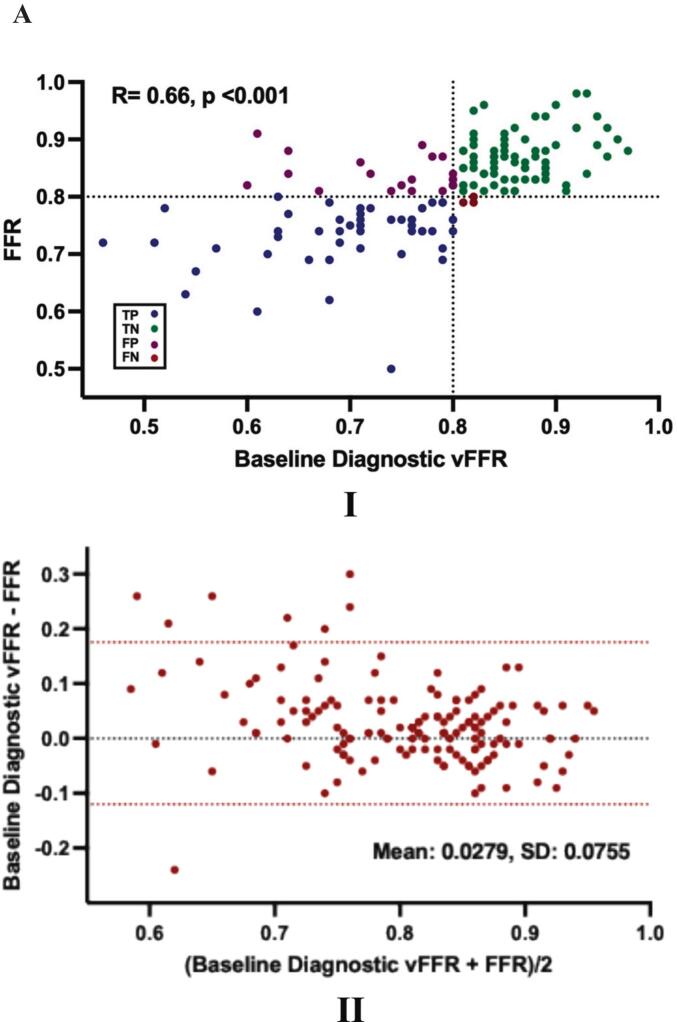

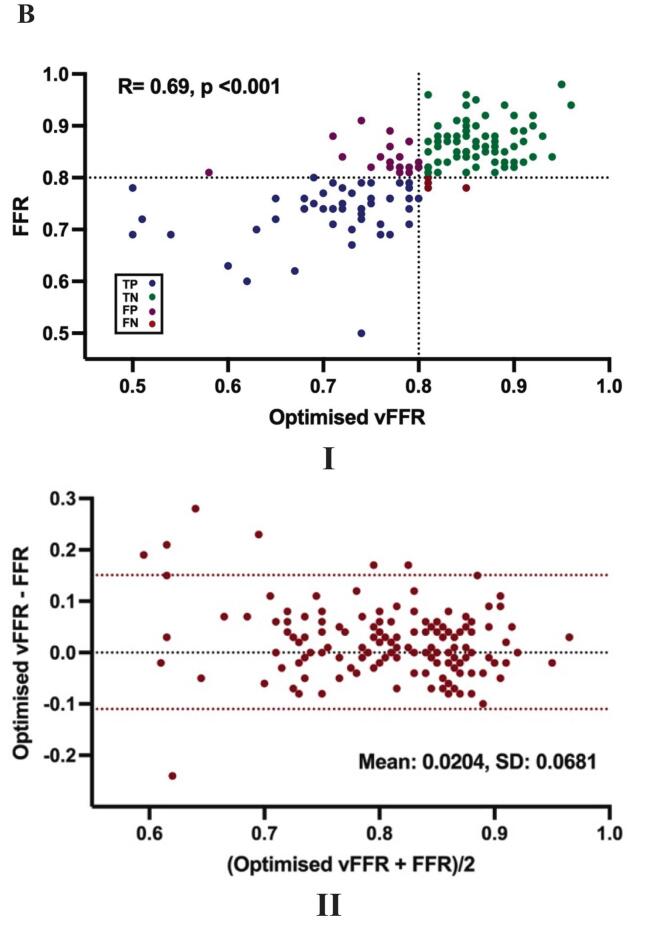
**Scatter and Bland-Altman Plots. (A). Baseline Diagnostic vFFR.** I. Scatter plot showing the relationship between Baseline Diagnostic vFFR and Wire-Based FFR with a vFFR threshold of ≤ 0.80. TP: True Positive, TN: True Negative, FP: False Positive, FN: False Negative. II. Bland-Altman plot of differences against the means. The mean bias is represented by the dashed black line and the 95% CI is represented by the dashed red lines. **(B). Optimised vFFR.** I. Scatter plot showing the relationship between Optimised vFFR and Wire-Based FFR with a vFFR threshold of ≤ 0.80. TP: True Positive, TN: True Negative, FP: False Positive, FN: False Negative. II. Bland-Altman plot of differences against the means. The mean bias is represented by the dashed black line and the 95% CI is represented by the dashed red lines. (For interpretation of the references to colour in this figure legend, the reader is referred to the web version of this article.)

**Fig. 3:**
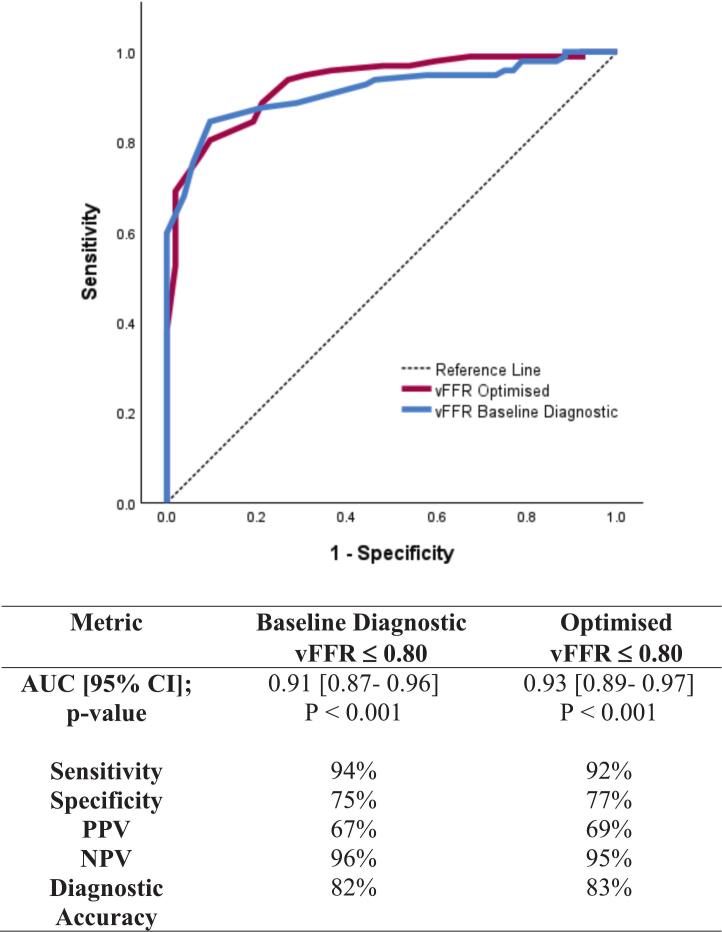
**vFFR Receiver Operating Characteristic (ROC) curve and Diagnostic Performance. Receiver Operating Characteristic (ROC) curve** for baseline diagnostic and optimised vFFR compared to wire-based FFR at a threshold of ≤ 0.80. **Abbreviations**: AUC: Area Under the Curve, NPV- Negative Predictive Value, PPV- Positive Predictive Value.

The mean baseline diagnostic vFFR was 0.79 ± 0.10, with values ≤ 80 observed in 49% of measurements **[**Table 1**]**. A moderate correlation was found between baseline diagnostic vFFR and wire-based FFR (R = 0.66, p < 0.001) with a mean bias of 0.0279 ± 0.0755 **[**Fig. 2**A I, II].** Receiver operating characteristic (ROC) curve analysis revealed that vFFR had excellent accuracy in predicting a wire-based FFR of ≤ 0.80 (AUC 0.91; 95% CI: 0.87–0.96) **[**Fig. 3**].** A vFFR threshold of ≤0.80 had a sensitivity of 94% (49/52), specificity of 75% (73/97), PPV of 67% (49/73), NPV of 96% (73/76) and diagnostic accuracy of 82% (122/149) **[**Fig. 3**].** Additional sub-group analysis across specific coronary vessel and patient subsets revealed consistent correlation and AUC findings with corresponding sensitivity and specificity also reported. **[**Table 2**].** Patient and lesion characteristics for false-positive and true-negative cases are presented in Table 3. No statistically significant differences were observed between the two groups.

**Table 2:** Subgroup analysis of baseline diagnostic vFFR.

	**Pearsons R**	**AUC [95% CI], p-value**	**Sensitivity**	**Specificity**
**Left Anterior Descending Artery/ Diagonal**	0.65	0.89, [0.84–0.95], p < 0.001	96%	71%
**Right Coronary Artery**	0.48	0.95, [0.86–1], p < 0.001	50%	89%
**Left Circumflex Artery/ Obtuse Marginal**	0.55	0.86, [0.62–1.1], p < 0.01	100%	80%
**Type 2 Diabetes**	0.72	0.93, [0.86–1.0], p < 0.001	92%	65%
**Current/ Ex-Smoker**	0.64	0.94, [0.88–0.99], p < 0.001	93%	70%
**Current Smoker**	0.83	0.89, [0.81–1], p < 0.001	83%	88%
**Focal Lesion (< 20 mm)**	0.61	0.87 [0.76–0.96], p < 0.001	94%	73%
**Diffuse Lesion (≥20 mm)**	0.68	0.93 [0.87–0.98], p < 0.001	94%	77%
**Bifurcation Lesion**	0.60	0.88 [0.75–1], p < 0.001	90%	69%
**Moderate or severe calcification**	0.53	0.96 [0.90–1], p < 0.001	90%	69%
**Frame Rate < 15 fps**	0.59	0.87 [0.77–0.96], p < 0.001	83%	78%
**Frame Rate ≥ 15 fps**	0.68	0.92 [0.87–0.98], p < 0.001	100%	72%

**Abbreviations:** fps: frames per second.

**Table 3:** Comparison of Patient and Lesion Characteristics Between False-Positive and True-Negative Cases for Baseline Diagnostic vFFR.

**Demographics**	**False Positives** **N = 24 lesions**	**True Negatives** **N = 73 lesions**	**p Value**
Age, years, median (IQR)	67 (54–73)	65 (60–73)	0.82
Male gender, n (%)	13 (54)	51 (70)	0.16
Body Mass Index, kg/m^2^, mean ± SD	27 ± 5	29 ± 6	0.08

**Cardiovascular Risk Factors, n (%)**			
Hypertension	18 (75)	55 (75)	0.97
Dyslipidaemia	18 (75)	60 (82)	0.44
Type 2 Diabetes	11 (46)	20 (27)	0.09
Current Smoker	2 (8)	14 (19)	0.21
Previous Myocardial Infarction	2 (8)	7 (10)	0.85

**Lesion Characteristics, n (%)**			
Focal lesion (< 20 mm)	11 (46)	29 (40)	0.60
Diffuse lesion (**≥**20 mm)	13 (54)	44 (60)	0.60
Bifurcation lesion	4 (17)	9 (12)	0.59
Ostial lesion	1 (4)	5 (7)	0.64
Tortuous lesion	1 (4)	3 (4)	0.99
Moderate or severe calcification	3 (13)	9 (12)	0.98

p < 0.05 was considered statistically significant.

#### 3.3.3 Optimised vFFR

The mean optimised vFFR was 0.80 ± 0.09, with values ≤ 80 observed in 47% of measurements **[**Table 1**].** A moderate correlation was found between optimised vFFR and wire-based FFR (R = 0.69, p < 0.001) with a mean bias of 0.0204 ± 0.0681 **[**Fig. 2**B I, II].** Receiver operating characteristic (ROC) curve analysis revealed that vFFR had excellent accuracy in predicting a wire-based FFR of ≤ 0.80 (AUC 0.93; 95% CI: 0.89–0.97) **[**Fig. 3**].** A vFFR threshold of ≤ 0.80 had a sensitivity of 92% (48/52), specificity of 77% (75/97), PPV of 69% (48/70), NPV of 95% (75/79) and diagnostic accuracy of 83% (123/149) **[**Fig. 3**].**

### 3.4 Comparison between baseline diagnostic and optimised vFFR

Baseline diagnostic and optimised vFFR were strongly correlated (R = 0.87, p < 0.001), with a mean bias of −0.0075 ± 0.0490 and an intraclass correlation coefficient of 0.93 (95% CI, 0.90–0.95), indicating excellent agreement. The AUCs were similar between baseline non-optimised and optimised vFFR (0.91 vs 0.93, p = 0.36).

## 4 Discussion

The VERMONT Non-Optimised study was a pre-specified subgroup analysis that extends our prior validation of vFFR by demonstrating that reliable physiological assessment with real-time vFFR is achievable using judiciously selected baseline diagnostic catheter images [7]. In this prospective, real-world cohort, baseline diagnostic vFFR showed excellent diagnostic performance against wire-based FFR, with high sensitivity (94%), strong negative predictive value (96%), and robust overall accuracy (AUC 0.91). Optimised vFFR performed similarly (AUC 0.93), with excellent agreement between the two approaches (ICC 0.93). These findings suggest that when baseline angiographic quality is sufficient, vFFR can function as a reliable first-line physiological screening tool, allowing invasive pressure-wire assessment to be reserved for vFFR positive cases.

The current findings are consistent with the FAST series, which first established the diagnostic accuracy of vFFR, and with our recent VERMONT study [4], [5], [6], [7]. FAST I and FAST EXTEND, based on retrospective baseline angiograms, reported high exclusion rates (63–68%), whereas FAST II and VERMONT, using prospectively optimised acquisitions, achieved much lower exclusion rates (9–15%) with excellent accuracy (AUC 0.91–0.93) [4], [5], [6], [7]. The present study bridges these approaches by prospectively assessing both baseline diagnostic and optimised imaging in routine practice. Compared with FAST II, rule-out performance was higher in the current study at the expense of lower rule-in specificity, reflecting a higher-risk cohort with greater prevalence of diabetes, smoking, and complex anatomy (diffuse, calcified, and bifurcation disease), which likely increases contouring difficulty and risk of false-positive vFFR [6], [7]. Despite these differences, both studies demonstrated similarly high overall accuracy (AUC 0.91–0.93), confirming the robustness of vFFR across prospective and retrospective settings using either optimised or judiciously selected baseline diagnostic images [6].

This study also highlights the central importance of angiographic image quality in vFFR computation. Most angiography-derived FFR platforms use computational fluid dynamics requiring at least two high-quality end-diastolic projections to permit accurate 3D reconstruction [11], [12]. The modelling heavily depends on reliable depiction of luminal geometry such as cross-sectional area, minimal lumen diameter, lesion length, and percentage stenosis [11], [12]. Current recommendations emphasise acquisition at ≥15 frames per second, with good vessel opacification, minimal overlap or foreshortening, and avoidance of panning. Angiograms acquired at 7 frames per second have reduced temporal resolution, which may limit precise identification of the end-diastolic frame, particularly in patients with higher heart rates. At higher heart rates, the shortened cardiac cycle results in fewer frames per cycle, increasing the likelihood that true end-diastole is not captured. Consequently, frames selected for analysis may be slightly offset from end-diastole, potentially introducing minor inaccuracies in vessel contour delineation and three-dimensional reconstruction.

The exclusion rate in this study (27.3%) reflects the practical challenges of maintaining optimal baseline diagnostic imaging in routine practice, yet vFFR remained accurate when baseline images were judiciously selected, even though 46% of baseline images were acquired at <15 frames per second. Appropriate selection of suitable baseline diagnostic images can therefore ensure reliable analysis. Furthermore, greater emphasis on optimising baseline angiographic technique is warranted to enhance the retrospective utility of angiography-derived FFR and emerging angiography based microvascular indices [13]. Novel single-projection approaches such as uFR (Pulse Medical) may further expand feasibility, particularly in retrospective analyses [14].

The ability to compute vFFR from baseline diagnostic angiogram images offers important clinical advantages. Prospectively, it may reduce contrast use, radiation exposure, and procedural time by avoiding the need for additional acquisitions which is particularly valuable in patients with renal impairment or high contrast burden. Retrospectively, vFFR may be applied to archived angiogram images to provide physiological insight for revascularisation decisions, offering particular value in the heart team setting without the need for repeat invasive testing. As a screening tool, baseline diagnostic vFFR in this study demonstrated strong rule-out performance, indicating that a negative result on routine baseline images may be sufficient to defer intervention safely. Whereas a positive result should undergo confirmatory wire-based testing. This pragmatic application could facilitate broader integration of physiology into everyday practice, even when optimised image sets are not available.

## 5 Limitations

This study has several limitations. First, it was conducted at a single centre with a modest sample size, which may limit the generalisability of the findings. Larger multicentre studies are needed to confirm the reproducibility of these results across diverse practice settings and operators. Second, vFFR analyses were performed by two experienced investigators rather than by an independent core laboratory. Although this reflects routine practice, it may introduce operator bias, particularly with manual contour adjustments.

Third, 27.3% of lesions were excluded due to inadequate baseline diagnostic image quality. This exclusion rate was substantially lower than that reported in the FAST I and FAST EXTEND studies (63–68%), which similarly relied on baseline diagnostic angiograms. Furthermore, the exclusion decisions were made by consensus between two investigators prior to vFFR analysis to limit bias. Despite this, the exclusion rate highlights the inherent selection bias associated with including only analysable cases and underscores the critical importance of image quality in angiography-derived physiological assessment. Notably, 54% of the included baseline diagnostic angiograms were acquired at a frame rate of ≥15 frames per second, which is higher than typically encountered in routine clinical practice. As such, the study cohort may not be fully representative of real-world practice and may be biased towards improved diagnostic performance. These factors may have influenced both the feasibility and diagnostic accuracy of vFFR and may limit the generalisability of the findings to an unselected population. Nevertheless, the results highlight the potential utility of baseline diagnostic catheter images for angiography-derived physiological assessment.

Fourth, most lesions analysed were located in the left anterior descending artery (79%), which may limit the generalisability of these findings to right coronary or left circumflex/obtuse marginal lesions. Fifth, analyses were conducted using a single software platform, which may limit applicability to other angiography-derived FFR systems.

Finally, this non-randomised study did not evaluate the impact of baseline diagnostic vFFR-guided decision making on clinical outcomes, as all revascularisation decisions were based on wire-derived FFR. Future multi-centre, randomised, outcome-driven trials are required to address this question.

## 6 Conclusions

In summary, the VERMONT Non-Optimised study demonstrated that real-time vFFR derived from judiciously selected baseline diagnostic angiogram images provided strong overall accuracy and high sensitivity for detecting physiologically significant lesions, with similar diagnostic performance to vFFR derived from optimised images. However, the modest exclusion rate (27.3%) highlights the dependence of angiography-derived physiological assessment on adequate image quality and reflects the potential selection bias inherent in analysing only suitable angiograms. When baseline angiographic quality is sufficient, these findings support the use of vFFR as a reliable first-line screening tool for intermediate lesions, reserving invasive pressure-wire assessment for positive cases. This approach may be particularly valuable prospectively in reducing contrast and radiation exposure and in enabling retrospective physiological assessment, particularly in a heart team setting.

## Conflict of interest statement

This investigator-initiated study received no industry-sponsored funding. Ethical approval was granted by the South Western Sydney Local Health District Human Research Ethics Committee (Study Number: 2021/ETH12209), and the study was conducted in accordance with the National Statement on Ethical Conduct in Human Research. A full waiver of consent was granted. All authors declare no relevant conflicts of interest related to this manuscript.

## CRediT authorship contribution statement

**Daniel Akrawi:** Writing – review & editing, Writing – original draft, Validation, Project administration, Methodology, Investigation, Formal analysis, Data curation, Conceptualization. **Krishna Kadappu:** Writing – review & editing, Supervision, Investigation, Conceptualization. **James Xu:** Writing – review & editing, Supervision, Conceptualization. **Tamer Yousef Naguib Badie:** Writing – review & editing, Methodology, Conceptualization. **Oliver Gibbs:** Writing – review & editing, Methodology. **Hashim Kachwalla:** Writing – review & editing, Methodology, Investigation, Conceptualization. **Phong TD Nguyen:** Methodology, Investigation, Conceptualization. **Rahul Kurup:** Writing – review & editing, Methodology, Investigation. **Upul Premawardhana:** Writing – review & editing, Supervision, Conceptualization. **Sidney Lo:** Writing – review & editing, Supervision. **Justyn Huang:** Writing – review & editing, Conceptualization. **Hao Tran:** Writing – review & editing, Resources. **Kavie Soosapilla:** Writing – review & editing, Methodology, Investigation. **Aiden O’loughlin:** Writing – review & editing, Supervision, Conceptualization. **Annemarie Hennessy:** Writing – review & editing, Supervision. **Giuseppe Femia:** Writing – review & editing, Supervision, Methodology, Investigation, Data curation, Conceptualization.

## Declaration of competing interest

The authors declare that they have no known competing financial interests or personal relationships that could have appeared to influence the work reported in this paper.

## Figures and Tables

**Table:** 

**Exclusion Criteria**	**Definition**
**Extensive vessel overlap**	Superimposition of another coronary vessel or structure obscuring the lesion or obscuring ≥ 50% of the target vessel.
**Foreshortening**	Angiographic projection resulting in incomplete visualisation of the lesion or ≥ 20% apparent shortening of the target vessel.
**Poor contrast opacification**	Inadequate contrast filling of the target vessel resulting in poorly defined vessel borders and inability to delineate the lumen along the target vessel.
**Excessive panning**	Continuous table movement during contrast injection preventing stable visualisation of the target vessel for ≥ 1 cardiac cycle.
**Inadequate angle projections**	Absence of two angiographic projections of the target vessel separated by ≥ 30 degrees.
